# Effect of Laser Posterior Nasal Neurolysis for the Treatment of Chronic Rhinitis: A Randomized Controlled Trial

**DOI:** 10.1002/alr.70133

**Published:** 2026-03-05

**Authors:** Jyun‐Yi Liao, En‐Ying Wang, Ying‐Shuo Hsu, Ming‐Shao Tsai, Cheng‐Jung Wu, Chia‐Hao Chang, Yi‐Li Hwang, Han‐Lo Teng, Jun‐Wei Hsieh, Chien‐Yu Huang

**Affiliations:** ^1^ Department of Otolaryngology‐Head and Neck Surgery Ditmanson Medical Foundation Chia‐Yi Christian Hospital Chiayi Taiwan, ROC; ^2^ Department of Otolaryngology Chiayi Hospital Ministry of Health and Welfare Chiayi Taiwan, ROC; ^3^ Department of Otolaryngology‐Head and Neck Surgery Shin Kong Wu Ho‐Su Hospital Taipei Taiwan, ROC; ^4^ Institute of Brain Science National Yang Ming Chiao Tung University Taipei Taiwan, ROC; ^5^ Sleep Research Center National Yang Ming Chiao Tung University Taipei Taiwan, ROC; ^6^ School of Medicine Fu Jen Catholic University New Taipei City Taiwan, ROC; ^7^ Department of Otolaryngology‐Head and Neck Surgery Chiayi Chang Gung Memorial Hospital Chiayi Taiwan, ROC; ^8^ Department of Otolaryngology School of Medicine College of Medicine Taipei Medical University Taipei Taiwan, ROC; ^9^ Department of Otolaryngology Shuang Ho Hospital Taipei Medical University Taipei Taiwan, ROC; ^10^ Department of Otolaryngology Lotung Poh‐Ai Hospital Luodong Township Yilan Taiwan, ROC; ^11^ Department of Oral and Maxillofacial Surgery Chia‐Yi Chang Gung Memorial Hospital Puzi Taiwan, ROC; ^12^ College of Artificial Intelligence National Yang‐Ming Chiao Tung University Tainan Taiwan, ROC

**Keywords:** laser, posterior nasal nerve neurolysis, rhinitis

## Abstract

**Background:**

To determine the safety and efficacy of laser ablation of the posterior nasal nerve (PNN) for the treatment of chronic rhinitis.

**Methods:**

This study was a single‐center, prospective, single‐blinded, randomized sham‐controlled trial. Patients with a 24‐h reflective Total Nasal Symptom Score (rTNSS) ≧ 5, rhinorrhea ≧ 2, and congestion ≧ 1, were randomized 2:1 to active PNN treatment with a CO_2_ laser device or a sham procedure. Outcome measures included the rTNSS, Nasal Obstruction Symptom Evaluation (NOSE), Pittsburgh Sleep Quality Index (PSQI), and Epworth Sleepiness Scale (ESS). The primary endpoint was the change in scores at 3 months.

**Results:**

Patients had a mean baseline rTNSS of 8.5 (95% CI, 7.9–9.1) and 8.2 (95% CI, 7.4–8.9) (*p* = 0.589) in the active treatment (*n* = 43) and sham control (n = 22) arms, respectively. At 3 months, the active treatment arm had a significantly greater decrease in rTNSS −4.7 (95% CI, −5.5 to −3.9) versus −2.6 (95% CI, −3.4 to −1.8) (*p* = 0.002). While the responder rate (RR, defined as ≧30% improvement rTNSS) was not significantly higher in the active treatment arm (81.4% vs. 63.6%, *p* = 0.119), post‐hoc analysis of RR (≧ 50% improvement) showed a significantly higher rate of 65.1% versus 31.8% (*p* = 0.011). There were greater improvements in the PSQI and ESS scores for the active arm over the sham arm at follow‐ups. (*p = *0.041 and 0.005, respectively).

**Conclusions:**

The CO_2_ laser posterior nasal neurolysis of the PNN area is associated with minimal adverse events and is superior to a sham procedure in reducing the symptom burden of chronic rhinitis.

## 1 Introduction

Chronic rhinitis is a debilitating condition that significantly impacts patients' overall well‐being. While rhinorrhea and sneezing are the most visible symptoms, the associated nocturnal nasal obstruction is a key driver of sleep disturbances [1]. Research consistently demonstrates a strong correlation between the severity of rhinitis and deteriorating sleep quality [2]. This lack of restorative sleep manifests as excessive daytime sleepiness, creating a vicious cycle of fatigue and reduced functional status. Recognizing and treating these sleep‐related sequelae is, therefore, essential for the comprehensive management of chronic rhinitis.

Surgical management for refractory rhinitis includes septomeatoplasty, inferior turbinate reduction, vidian neurectomy, and posterior nasal nerve neurolysis (PNNN) [3, 4, 5]. Recently, the focus has shifted toward either cryotherapy or temperature‐controlled radiofrequency PNNN [6, 7, 8] to address the limitations of earlier techniques. While Vidian nerve resection is effective, it poses risks of dry eye and numbness of the palate [9, 10]. PNNN offers a more selective denervation strategy, aiming to maximize symptom control while minimizing collateral complications and the bleeding risks associated with extensive turbinate resection.

Although PNNN is endorsed by major otolaryngology societies [11, 12], the current literature highlights notable clinical and technical limitations. Clinically, specific concerns regarding delayed symptom relief and hemorrhagic risks persist. Initial response rates are suboptimal (67.5%–76.2% at 3 months) [13, 14, 15], and major posterior nasal bleeding occurs in approximately 2% of cases, often as a delayed complication [16]. While most FDA‐approved PNNN devices rely on contact‐based mechanisms, the bulky designs and fixed angles of touch surfaces have limited their utility in targeting these branches within patients with narrowed nasal anatomy. These findings suggest a pressing need for studies utilizing diverse surgical instruments to confirm whether different modalities can improve safety and treatment outcomes.

In contrast to bulky contact‐based devices, laser technology offers a safe and precise alternative. The small caliber of the laser fiber allows for pinpoint accuracy in mapping and ablating the PNN, even within confined anatomical spaces. Krespi et al. [17] demonstrated this potential, reporting a favorable safety profile with no serious complications and a satisfying 62% reduction in rTNSS after 90 days using a diode laser. Hwang et al. [18] reported that combining radiofrequency turbinoplasty with laser PNNN can be performed safely under local anesthesia, resulting in high response rates (100%) with low complications in a 6‐month follow‐up. Liao et al. [19] reported adding laser PNNN to turbinate surgeries when treating refractory rhinitis medicamentosa, which enhanced control of nasal itching and achieved earlier responses in rhinorrhea and sneezing control. Despite these advantages and the widespread availability of lasers in ENT departments globally, no randomized controlled trial (RCT) has yet been conducted to rigorously validate the safety and efficacy of laser PNNN.

With this background, we hypothesized that laser ablation of the posterior nasal nerve (LPN3) may provide significant improvement in chronic rhinitis symptoms with a superior safety profile. The objective of this study was to determine the safety and efficacy of the LPN3 in a RCT with a sham procedure as the control arm. In this report, we detail the primary endpoint at the 3‐month follow‐up.

## 2 Materials and Methods

### 2.1 Study Design and Patient Population

This was a prospective, single‐center, single‐blinded RCT with a sham procedure control arm. The trial was a superiority design with a one‐way crossover available to the patients randomized to the sham control arm after their 3‐month follow‐up visit, if still eligible. Data from crossover patients will be available for future publications. Patients were enrolled at Chia‐Yi Christian Hospital, and all interventions were performed by the senior author (C.Y.H.). This study was approved by the Ethics Committee of the Chia‐Yi Christian Hospital (No: IRB2024071). The trial was registered at www.clinicaltrials.gov with the unique identifier NCT07050992.

Eligible participants were adults aged 18–65 years diagnosed with chronic rhinitis that had persisted for at least 6 months and proved refractory to medical management, defined as a lack of sustained improvement despite the use of intranasal corticosteroids combined with intermittent oral antihistamines or on‐demand combination nasal sprays. Ipratropium bromide nasal spray was neither prescribed nor evaluated, as it is not commercially available in the study region. Inclusion required a baseline 24‐h reflective Total Nasal Symptom Score (rTNSS) of five or higher, characterized explicitly by moderate‐to‐severe rhinorrhea (Subscore 2–3) and mild‐to‐severe nasal congestion (Subscore 1–3), alongside confirmation of the absence of significant rhinosinusitis via nasal endoscopy or computed tomography within the preceding month. Candidates were excluded if they presented with obstructive anatomical abnormalities or postsurgical alterations limiting endoscopic access to the posterior nasal passages, active sinonasal infection, or a history of severe xerophthalmia, chronic epistaxis, rhinitis medicamentosa, or head and neck radiotherapy. Further exclusion criteria included a known bleeding diathesis, current use of anticoagulants that could not be discontinued, prior surgical interventions specifically for chronic rhinitis, or a documented history of compromised wound healing following head and neck surgery.

Before enrollment, all patients underwent a comprehensive medical and medication history review, routine endoscopy, and a home sleep test (HST) to evaluate for obstructive sleep apnea (OSA). Baseline symptom severity was assessed using validated questionnaires, including the rTNSS, Nasal Obstruction Symptom Evaluation (NOSE), Pittsburgh Sleep Quality Index (PSQI), and Epworth Sleepiness Scale (ESS). Participants were subsequently randomized to the active treatment or sham control arm in a 2:1 ratio using a pre‐generated random number sequence. To ensure blinding, identical patient preparation, including the administration of topical anesthesia and local infiltration anesthesia, was performed in both study arms.

### 2.2 Procedure

The LPN3 procedure was performed in an office‐based setting under local anesthesia. Nasal decongestion was achieved by placing cotton pledgets in the bilateral inferior and middle meatuses for 5 min. Following the decongestion of the inferior turbinate, the patient was brought to the test chair with their eyes blindfolded, preparing for rigid nasoendoscopy examination.

To avoid the risk of future posterior major epistaxis, we developed a routine technique called “posterior lateral nasal artery (PLNA) detection.” Before further local anesthesia, a 2.9 mm, 0‐degree rigid endoscope was used to examine the middle meatus and identify the course of the PLNA. The endoscope was maintained stationary near the middle turbinate for 15 to 30 s to visualize arterial pulsation directly on the lateral nasal wall mucosa. In the majority of cases, the PLNA, which originates from the sphenopalatine artery, can be identified by its pulsation beneath the mucosa of the lateral nasal wall. This landmark was typically located approximately 0.5–1 cm anterior to the posterior end of the middle turbinate. The arterial course generally presents a horizontal segment emerging from the sphenopalatine artery, followed by a vertical descending segment with 1 to 2 terminal branches that enter the inferior turbinate. In cases of significant mucosal edema due to severe allergic reactions or chronic congestion, additional decongestion of the lateral nasal wall was performed using cottonoids soaked in epinephrine, which could enhance vessel pulsation and help identify the artery route. Upon identification of the PLNA, the anterior and posterior margins were defined as 2–3 mm anterior and posterior to the vessel's course as a guide for a safe zone for later laser ablation. Crucially, this vessel identification process was performed before laser ablation, as the subsequent mucosal tightening effect by thermal energy could diminish visible pulsations and obscure the vessel's location.

Once the vessel was localized, approximately 1 mL of 2% lidocaine (without epinephrine) was injected at the insertion of the middle turbinate into the lateral nasal wall to infiltrate the posterior nasal nerve region. A period of 1 min was allowed for anesthetic onset before patients were treated according to their randomization group.

**FIGURE 1:**
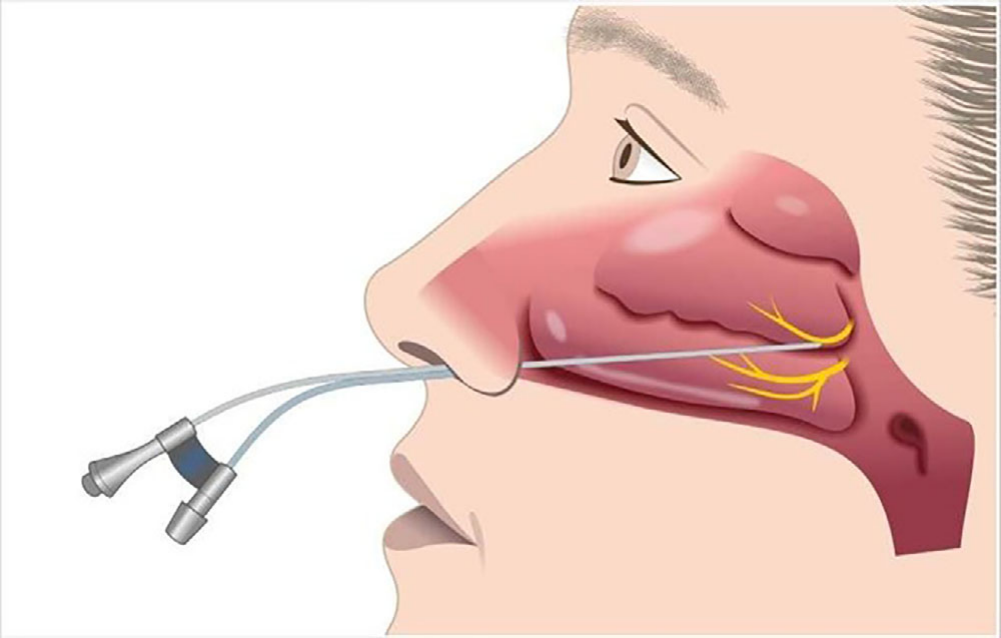
Posterior nasal nerve ablation with CO_2_ laser. The posterior lateral nasal artery was identified on the lateral nasal wall. Careful visualization enabled the identification and avoidance of vessel injury during laser ablation. The laser was transmitted via a nasal probe angulated using a straight‐tip 90° mirror.

For the active treatment group, PNNN was performed using a 2‐W continuous‐wave laser with the AcuPulse CO_2_ laser (Lumenis Ltd., Yokne'am Illit, Israel), transmitted via a nasal probe angled using a straight‐tip 90 ° mirror. (Figure 1). First, we ablate the local anesthesia injection point over the middle turbinate as a hemostasis measure. Then, we use the CO_2_ laser to outline the anterior and posterior margins of the PLNA route, as identified previously, to prevent inadvertent injury to the vessel. The PNN ablation area includes the posterior margin of the PLNA artery to the middle turbinates end through the lateral nasal wall till the superior aspect of the Eustachian tube, and also the anterior margin of the PLNA artery to the lateral nasal wall, where the CO_2_ laser could ablate up to 1.5 cm anterior to the anterior margin. The posterior 1 cm of the medial‐superior portion of the inferior turbinate was also ablated. The laser ablation was performed until visible mucosal whitening and a brownish charring were observed on the lateral nasal wall. This procedure took approximately 2–3 min/side.

For the sham surgery group, the same laser surgical instrument was used to enter the nasal cavity. The energy setting utilizes the lowest limit of 0.5 W with intermittent emission (0.01‐s emission with a 1‐s rest), and the emission mode is set to Super Pulse to minimize heat dispersion. The overall delivered energy effect of the sham surgery on the mucosa was near undetectable after the intervention. During the sham surgery, intermittent gentle compression of the nasal probe tip over the mucosal surface at the lower edge of the middle turbinate, posterior superior aspect of the inferior turbinates, and posterior choana for 2 min on each side. This is to produce the sound, light, and tactile effects of the laser intervention, but without sufficient thermal energy transfer to cause detectable mucosal changes and tissue alterations, serving as a high‐quality sham surgery control group.

Nasal packing was never used, as all patients had a patent airway upon completion of the procedure. Postoperative care included 3 days of antihistamines, antibiotics, and analgesics. No nasal irrigation or rinsing was necessary. The patients were followed up at 1 week, 1 month, and 3 months, with no additional antihistamine or nasal spray prescribed during the follow‐up visits.

### 2.3 Surveys

Patients were evaluated using the 24‐h rTNSS, the NOSE scale [20, 21] to assess preoperative and postoperative nasal symptoms and quality of life. The 24‐h rTNSS is a four‐item questionnaire with a total score ranging from 0 to 12. The questionnaire focuses on the patient's recent symptoms of nasal congestion, rhinorrhea, itching, and sneezing within the past 24 h. The NOSE scale is a nasal symptom‐related quality‐of‐life questionnaire composed of five items, with a total score ranging from 0 to 100, reflecting the quality‐of‐life restrictions in patients with nasal problems. Sleep quality was evaluated using the PSQI [22], and daytime sleepiness related to poor sleep quality was assessed using the ESS [23]. A numerical rating scale (NRS) for verbalizing pain severity (0–10) was used, where 0 indicated no pain at all, and 10 represented the worst pain one could imagine, during both local anesthesia injection and active or sham ablation. The ratings were recorded immediately after the procedure. Postoperative side effects and complications were documented during each follow‐up visit.

### 2.4 Outcome Evaluation

The primary endpoint was the change in the rTNSS score from baseline to the 3‐month follow‐up, as well as the responder rate, defined as a ≥ 30% reduction in rTNSS from baseline. Secondary endpoints included the changes in NOSE, sleep quality (as measured by the PSQI), and daytime sleepiness (as measured by the ESS). Safety outcomes, including device‐ and procedure‐related serious adverse events and postoperative complications, were assessed through 3 months. Although this report focuses on short‐term outcomes, the trial protocol includes a planned one‐year follow‐up.

### 2.5 Statistical Analysis

Descriptive statistics for continuous variables were presented as means with standard deviations (SD) for normally distributed data, or medians with interquartile ranges (IQR) for non‐normally distributed data. Categorical variables were summarized as frequencies and percentages. Baseline demographic characteristics were compared between the active treatment and sham control arms using independent *t*‐tests and Fisher's exact tests. To evaluate treatment outcomes, we calculated 95% confidence intervals (CI) for changes from baseline. For non‐normally distributed continuous variables or small sample size groups (*n* < 30), 95% CI were estimated using the bootstrapping method with 2000 resamples and bias‐corrected and accelerated adjustment to ensure robustness. Within‐group changes were analyzed using paired *t*‐tests or Wilcoxon signed‐rank tests, depending on whether the data were normally distributed. Between‐group differences were assessed using independent *t*‐tests for parametric data, and Mann–Whitney *U* tests for non‐parametric data. All statistical tests were two‐tailed, with significance set at *p* < 0.05. Analyses were performed using PASW Statistics (version 18.0.0, SPSS Inc., Chicago, IL, USA).

The sample size estimation was based on the study by Stolovitzky et al. [24], which reported a mean improvement of −3.6 (95% CI, −4.2 to −3.0) in the active treatment group versus −2.2 in the control group. Based on these data, the estimated effect size (Cohen's *d*) was approximately 0.53. To achieve 80% statistical power with a 2:1 allocation ratio (active vs. sham) at a significance level of 0.05, an a priori calculation indicated a required sample size of approximately 128 participants. On anticipating a potentially larger effect size due to our strict inclusion criteria (which excluded revision cases), our protocol established a target of 120 subjects. However, due to the fixed 12‐month study duration and strict inclusion criteria (excluding revision cases), patient recruitment was concluded with 66 participants enrolled.

## 3 Results

**FIGURE 2:**
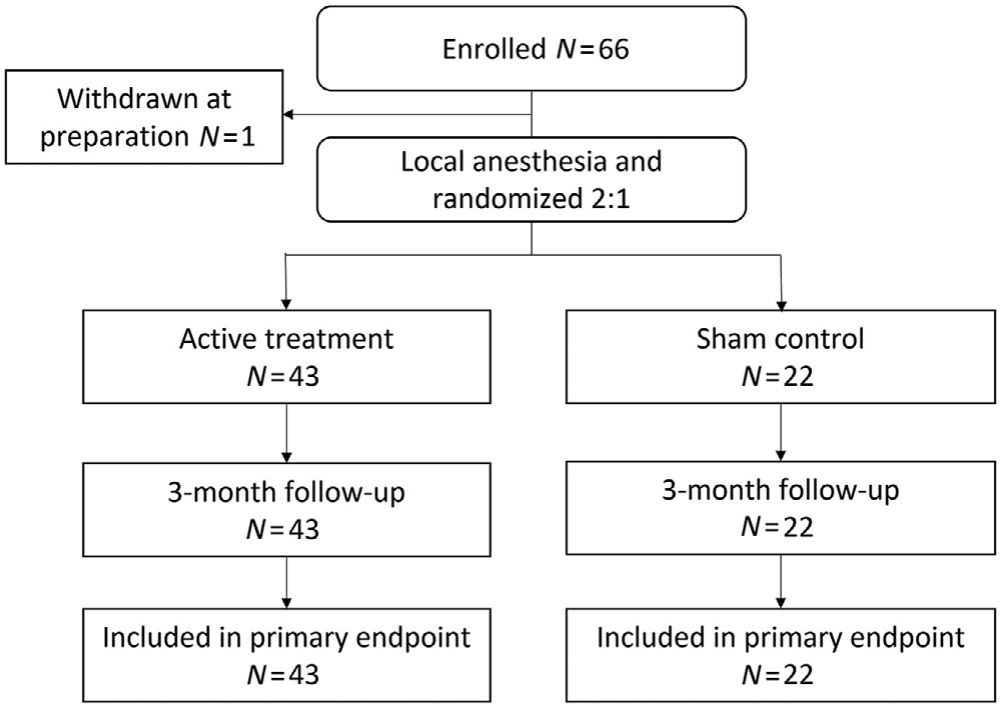
Enrollment, treatment arm allocations, and follow‐up through 3 months post procedure.

A total of 66 patients were enrolled, randomized, and treated between November 2024 and September 2025. Of these, 1 patient had severe sneezing after initial nasal packing with cottonoid and instrument manipulation over the middle meatus and was withdrawn before starting further procedures. Thus, 43 were assigned to active treatment, and 22 were assigned to the sham procedure control (Figure 2). All 65 patients who received intervention had completed the 3‐month follow‐up and were available for primary endpoint analysis at 3‐months. The basic demographics and baseline characteristics of the patients in each arm are presented in Table 1. The overall mean rTNSS in each arm was not significantly different at baseline: 8.5 ± 2.0 for the active treatment arm and 8.2 ± 1.8 for the sham control arm (*p* = 0.589). Medication use between the arms was not significantly different at baseline. The two groups had no significant difference in BMI, OSA, PSQI, ESS, and NOSE score. The NRS for pain during injection anesthesia was similar in both groups, 3.1 ± 2.0 versus 3.1 ± 2.3, *p* = 0.899, and procedure pain was higher in the active treatment group, 4.8 ± 2.2 versus 3.7 ± 2.4, *p* = 0.048, reflecting the actual energy delivery that caused additional pain sensation. However, all pain was tolerable, as it was a local anesthesia procedure.

**TABLE 1:** Patient demographics, baseline, and procedural information by treatment group.

Characteristic	Active treatment *n* = 43	Sham control *n* = 22	*p* value
Female sex	21 (48.8)	7 (31.8)	0.196
Age (years)	36.9 ± 9.3	38.0 ± 10.9	0.679
Body mass index	24.0 ± 3.7	23.8 ± 3.8	0.672
OSA	26 (60.4)	14 (63.6)	0.807
Mod‐severe	9 (20.9)	7 (31.8)	0.343
AHI	8.9 ± 8.3	11.8 ± 10.4	0.22
LoSaO2	86.0 ± 5.7	84.1 ± 5.8	0.213
PSQI	10.4 ± 4.1	9.1 ± 4.1	0.217
ESS	8.0 ± 3.9	7.6 ± 4.1	0.712
Asthma	1 (2.3)	2 (9.1)	0.225
Medication use			
Antihistamines	26 (60.5)	12 (54.5)	0.653
INCS	13 (30.2)	7 (31.8)	0.898
SLIT	1 (2.3)	0 (0)	0.479
rTNSS score	8.5 ± 2.0	8.2 ± 1.8	0.589
NOSE score	56.9 ± 24.4	57.7 ± 21.3	0.888
Pain NRS‐anesthesia	3.1 ± 2.0	3.1 ± 2.3	0.899
Pain NRS‐procedure	4.8 ± 2.2	3.7 ± 2.4	0.048

Abbreviations: AHI, apnea‐hypopnea index; ESS, Epworth Sleepiness Scale; INCS, intranasal corticosteroid spray; LoSaO2, lowest saturation of O2; Mod‐severe, moderate to severe disease; NOSE, Nasal Obstruction Symptom Evaluation; NRS, numerical rating scale; OSA, obstructive sleep apnea; PSQI, Pittsburgh Sleep Quality Index; rTNSS, reflective Total Nasal Symptom Score; SLIT, sublingual immune therapy.

**FIGURE 3:**
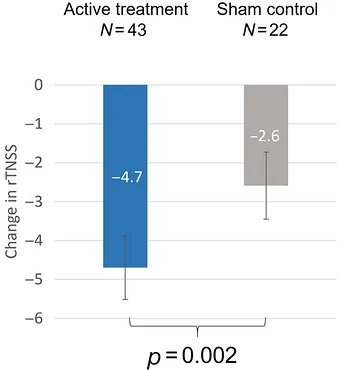
Primary endpoint at 3 months, change in reflective Total Nasal Symptom Score (rTNSS) from baseline. Change in active treatment arm was significantly greater than in sham control (*p* = 0.002). Bars represent 95% CIs.

**FIGURE 4:**
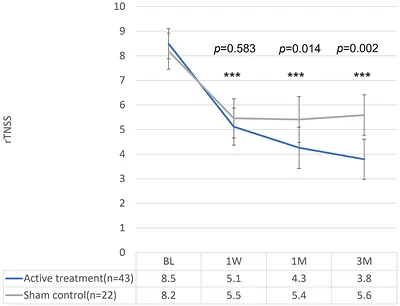
Reflective Total Nasal Symptom Score (rTNSS) during 3‐month follow‐up compared to baseline. The change in the active treatment arm was significantly greater than in the sham control from 1 month to 3 months (*p* = 0.022 and 0.002). ***indicates intra‐group 3‐month result compared to baseline in both groups, *p* < 0.001. Bars represent 95% CIs. 1M, 1 month; 1W, 1 week; 3M, 3 month; BL, baseline.

At 3 months, the mean rTNSS reduction was significantly greater in the active treatment group, −4.7 (95% CI, −5.5 to −3.9), than in the sham control group, −2.6 (95% CI, −3.4 to −1.8; *p* = 0.002) (Figure 3). A significant change from baseline was found in both groups when comparing the 3‐month follow‐up score to baseline (*p* < 0.001). This represents a 55.3% improvement in score from baseline in the active treatment arm versus a 31.7% improvement in the sham control arm. When comparing group differences, the active treatment group showed significantly greater change than the sham control group at both 1‐month (*p* = 0.022) and 3‐month (*p* = 0.002) follow‐up periods. (Figure 4)

**FIGURE 5:**
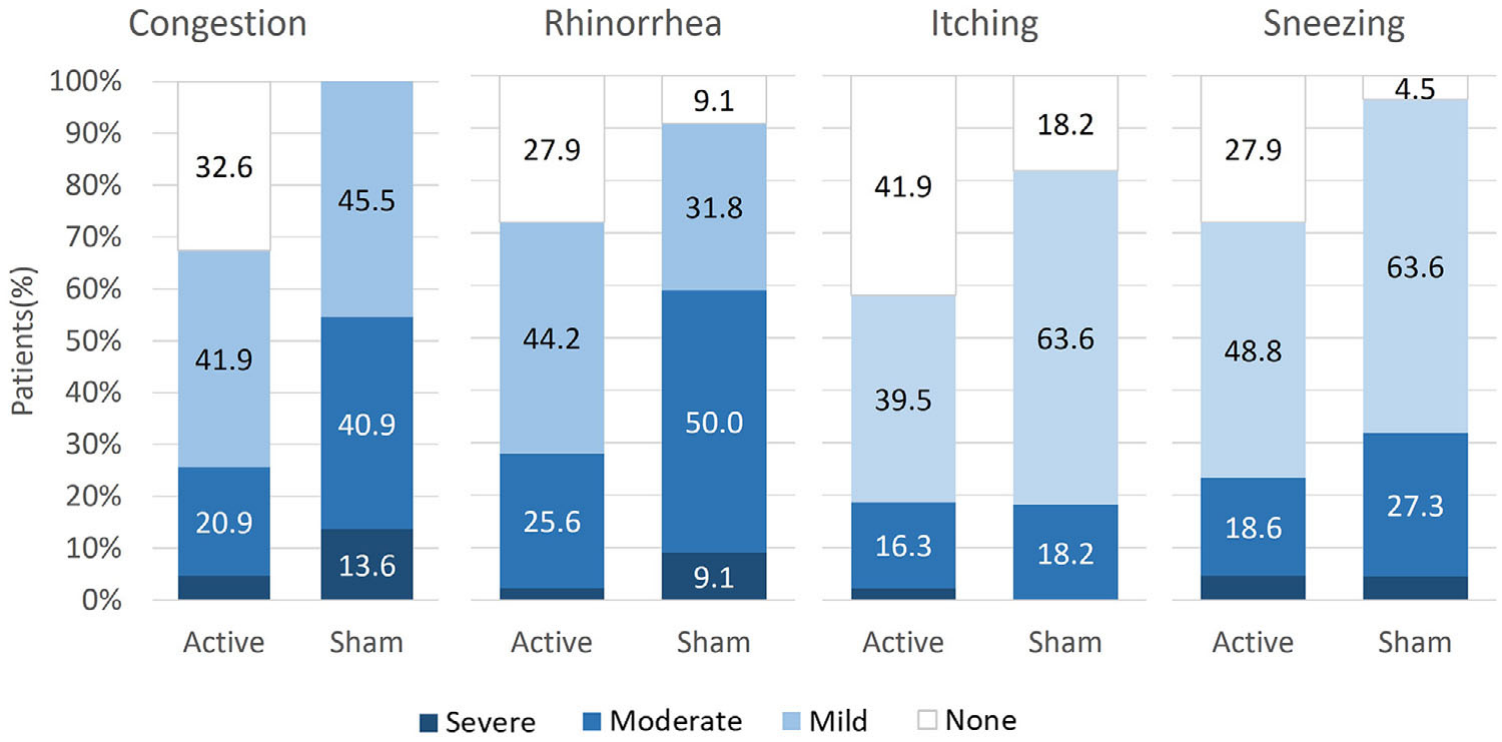
The distribution of patients with the different reflective Total Nasal Symptom Score (rTNSS) subscores at 3 months in the active treatment and sham control arms.

The decrease in rTNSS congestion and rhinorrhea subscores from baseline through 3 months was significantly greater in the active treatment arm than in the sham control arm (Table 2). The difference in decrease in rTNSS itching and sneezing subscore from baseline through 3 months between arms did not reach statistical significance (Table 2). The distribution of the percentage of patients reporting each subscore clearly shows the significantly larger shift toward lower subscores for rhinorrhea and congestion following active treatment (Figure 5).

**TABLE 2:** Reflective Total Nasal Symptom Score subscore at baseline and the change from baseline through 3 months in the active treatment and sham control arms.

	Active treatment (*n* = 43)				Sham control (*n* = 22)				
Characteristic	Mean	95% CI	Median	IQR	Mean	95% CI	Median	IQR	*p* value^a^
At baseline									
Congestion	2.3	2.1–2.5	2	2–3	2.3	2.0–2.6	2	2–3	0.928
Rhinorrhea	2.4	2.2–2.6	2	2–3	2.4	2.1–2.6	2	2–3	0.548
Itching	1.8	1.5–2.0	2	1–2	1.8	1.5–2.1	2	1–2	0.982
Sneezing	2.0	1.7–2.3	2	1–2	1.7	1.4–2.1	2	1–2	0.188
Change from baseline									
Congestion	1.0	0.7–1.2	1	0–2	1.7	1.4–2.0	2	1–2	0.002
Rhinorrhea	1.0	0.8–1.3	1	0–2	1.6	1.2–1.9	2	1–2	0.01
Itching	0.8	0.5–1.0	1	0–1	1.0	0.7–1.3	1	1–1	0.185
Sneezing	1.0	0.7–1.3	1	0–1	1.3	1.0–1.6	1	1–2	0.088

Abbreviation: IQR, interquartile range.

^a^ Compared by Wilcoxon–Mann–Whitney test.

**FIGURE 6:**
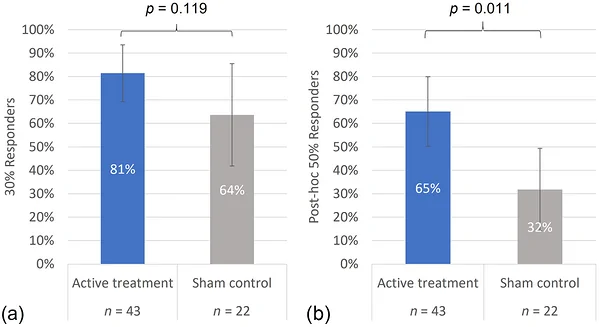
Primary endpoint of responder rates at 3 months. (a) Defined as a 30% improvement in reflective Total Nasal Symptom Score (rTNSS) from baseline (*p* = 0.119). (b) Post‐hoc exploratory analysis of 50% responders showed active treatment was superior to the sham procedure control (*p* = 0.011). Bars represent 95% CIs.

Although the 30% responder rate was numerically higher in the active treatment group, 81.4% (95% CI, 69.3%–93.5%) than in the sham group, 63.6% (95% CI, 41.8%–85.5%), this difference did not reach statistical significance (*p* = 0.119). Post‐hoc exploratory analysis using a threshold of ≧ 50% reduction in rTNSS from baseline, the responder rate was significantly higher in the active treatment arm (65.1%, 95% CI, 50.3%–80.0%) compared with the sham control arm (31.8%, 95% CI, 14.9%–50.0%), *p* = 0.011 (Figure 6).

For the NOSE score, the active treatment group showed a numerical decrease of −31.2 (95% CI, −39.2 to −23.1) compared with the sham control group, −24.5 (95% CI, −33.4 to −15.7) during the 3 months after the procedure; however, this result only trended towards a difference and did not reach statistical significance.(*p = *0.301)

**FIGURE 7:**
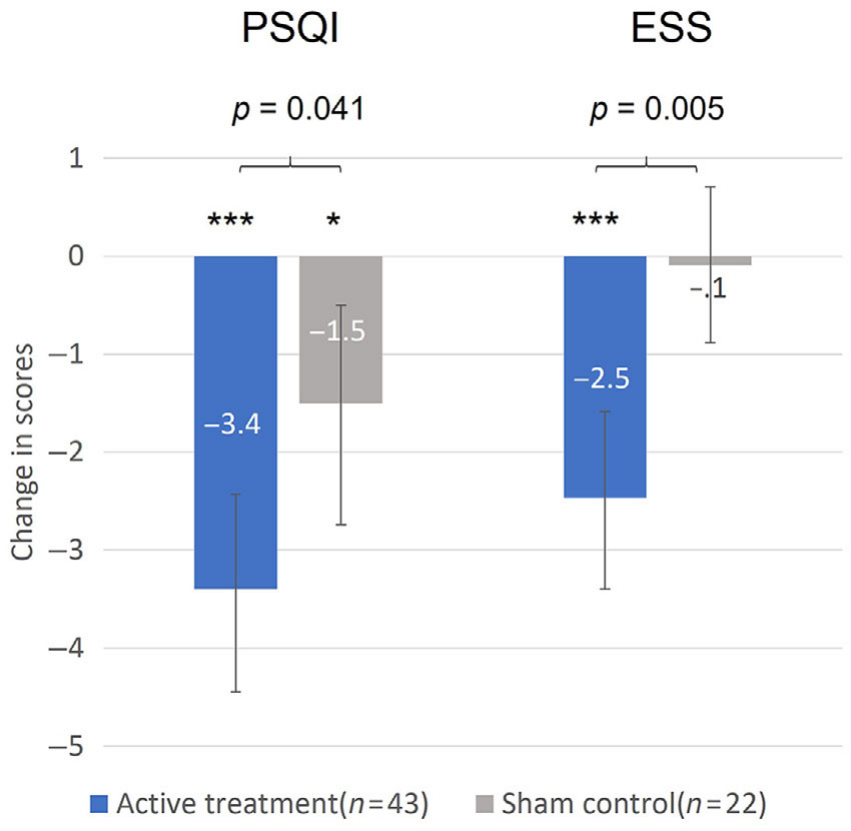
Secondary endpoint at 3 months, change in Pittsburgh Sleep Quality Index (PSQI) and Epworth Sleepiness Scales (ESS) score from baseline. The change in the active treatment arm was significantly greater than in the sham control (*p* = 0.041 and 0.005). *p < 0.05, ***p < 0.001 indicates intra‐group 3‐month result compared to baseline. Bars represent 95% CIs.

**FIGURE 8:**
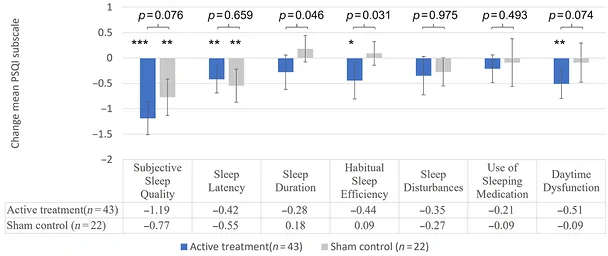
Secondary endpoint at 3 months. Pittsburgh Sleep Quality Index (PSQI) subscores change from baseline. The change in the active treatment arm was significantly greater than in the sham control in terms of sleep duration and habitual sleep efficiency. *p < 0.05, **p < 0.01, ***p < 0.001 indicates intra‐group 3‐month result comparing to baseline. The *p* value report in the graph indicates the assessment of between‐group differences. Bars represent 95% CIs.

For evaluation of sleep quality and daytime sleepiness, the active treatment group showed significant improvement in PSQI and ESS scores during the 3‐month follow‐up with PSQI change from 10.4 (95% CI 9.2–11.7) to 7.0 (95% CI 5.8–8.2) and ESS 8.0 (95% CI 6.8–9.2) to 5.6 (95% CI 4.5–6.6) (both *p* < 0.001) (Figure 7). Furthermore, a significantly greater decrease in mean PSQI and ESS in the active treatment group was −3.4 (95% CI, −4.5 to −2.3) and ‐2.5 (95% CI, −3.4 to −1.5), respectively, significantly more than the sham control group −1.5 (95% CI, −2.7 to −0.5) and ‐0.1 (95% CI, −0.9 to 0.7), respectively, during the 3 months after the procedure (*p* = 0.041 and 0.005, respectively). The PSQI subscores analysis (Figure 8) revealed a significant change(improvement) in subjective sleep quality, sleep latency, habitual sleep efficiency, and daytime dysfunction in the active treatment group and subjective sleep quality, sleep latency in the sham control group. When considering the group differences, sleep duration (*p* = 0.046) and habitual sleep efficiency (*p* = 0.031) scores changed when compared with the sham control group.

During the follow‐up period, one patient in the sham control group required an increase in medication dosage with additional regular use of an intranasal corticosteroid spray and was a non‐responder. All other patients reported no change or decrease in the use of chronic rhinitis medication compared to their baseline medication usage.

At the 1‐week follow‐up visit, a majority of patients in the active treatment group reported a sensation of tenacious mucus retention and nasal fullness, which typically began within days of the procedure. Patients described these secretions as viscous and difficult to evacuate; however, symptoms gradually subsided over the subsequent weeks as the mucosa healed. Regarding hemorrhagic events within the first month, three patients in the active group and five in the sham group experienced short‐term, self‐limited epistaxis within 3 days after the procedures. Notably, one episode in the active treatment group (Day 8) was triggered by intense crying but resolved spontaneously within 5 min without intervention. By the 3‐month endpoint, no major complications—such as significant posterior nasal bleeding, dry eye, headache, sinusitis, wound infection, or synechia—were observed.

## 4 Discussion

This study, to our knowledge, represents the first RCT evaluating the safety and efficacy of laser treatment in PNNN. Our results demonstrate that LPN3 provides rapid and significant symptom relief in patients with chronic rhinitis compared to a sham procedure. At the 3‐month follow‐up, the active treatment group showed statistically significant improvements in rTNSS, PSQI, and ESS scores. Notably, the procedure demonstrated an excellent safety profile, with no incidence of significant bleeding or permanent complications. We attribute this absence of hemorrhagic risks specifically to our intraoperative “PLNA detection” protocol, which enables visual identification and preservation of the artery before ablation.

Although the initial power analysis suggested a sample size of 120, the final analysis included 65 participants (43 in the active treatment group and 22 in the sham control group) following one withdrawal. Recruitment was limited by the fixed 12‐month study duration at a single center and the rigorous inclusion criteria. Unlike previous RCTs [24, 25], this study strictly excluded patients with prior nasal surgeries to ensure a homogeneous population of primary cases. Our initial sample size calculation (*N* = 120) was based conservatively on the study by Stolovitzky et al. [24], which reported a mean rTNSS change of −3.6. However, to obtain a more representative estimate of the treatment effect, we performed a post‐hoc pooled analysis incorporating data from four major studies: Stolovitzky et al. (mean −3.6; 95% CI, −4.2 to −3; *n* = 77) [24], Ehmer et al. (mean −5.1; 95% CI, −5.8 to −4.4; *n* = 47) [26], Lee et al. (mean −4.2; 95% CI, −4.6 to −3.7; *n* = 128) [27], and Davis et al. (mean −4.1; 95% CI, −4.8 to −3.5; *n* = 79) [28]. This comprehensive analysis yielded a weighted mean improvement of −4.16 with a pooled standard deviation of 2.65. When compared to the sham control mean of −1.91 [29], the revised effect size (Cohen's *d* = 0.85) indicates that a sample size of only 51 participants (34 active, 17 sham) would have been sufficient to achieve 80% power with a 2:1 allocation ratio. Our final analysis of 65 participants exceeds this requirement significantly. Furthermore, our study observed an effect size (Cohen's *d* = 0.79) consistent with those of the high‐impact studies, confirming that the current sample size provided robust statistical power (> 88%) to detect the intervention effect.

Current literature on PNNN, primarily utilizing cryotherapy or radiofrequency, reports variable early outcomes. Large cohort studies have indicated response rates of approximately 67.5%–76.2% at the 3‐month interval, often requiring up to a year to achieve maximal efficacy [14, 25, 27, 28, 30]. In our cohort, the 3‐month response rates were 81.4% using the ≧ 30% reduction a priori benchmark and 65.1% based on a ≧ 50% reduction post hoc exploratory analysis. These outcomes are comparable to the efficacy rates reported for RFA and cryoablation in existing literature, suggesting that LPN3 serves as a promising and viable alternative to established modalities. The current study also excluded individuals who had received any nasal surgery, making our results more robust and reliable.

A critical factor contributing to the superior safety and tolerability profile in our study was the implementation of a rigorous “PLNA detection” technique. The PLNA is the major branch of the sphenopalatine artery and courses downward at approximately 1 cm anterior to the posterior end of the middle turbinate and ends as the feeding artery of the inferior turbinates with a terminal branch or two divided branches [31]. The majority of the postganglionic nerves originating from the pterygopalatine ganglion typically course posterior to the PLNA [32], which could be a small region when turbinates are swollen. In conventional contact‐based ablation, the relatively bulky probes often obscure the surgical field, making it less likely to detect pulsation of mucosal vessels during lateral wall mucosa ablation, which can potentially lead to inadvertent thermal injury to the arterial wall and delayed bleeding. Unlike devices that obscure the surgical field, the non‐contact laser enables continuous visualization of PLNA pulsation even during firing, allowing us to clearly identify the artery and maintain a strict 2–3 mm safety margin during ablation. This precise “buffer zone” strategy potentially prevented the delayed hemorrhage by sparing the arterial wall. Furthermore, the laser eliminates mechanical trauma associated with probe retrieval (e.g., cryoadhesion) and offers titratable energy settings for thickened mucosa. Importantly, our outcomes confirm that preserving this non‐ablated area did not compromise surgical efficacy, demonstrating that targeted, PLNA route‐spared neurolysis is sufficient for symptom relief without the need for aggressive, confluent cauterization.

To the best of our knowledge, this is also the first study to specifically evaluate and validate the impact of office‐based PNNN on sleep quality (PSQI) and daytime sleepiness (ESS). Takabayashi et al. [33] reported that postoperative PSQI scores in the nasal surgery group (endoscopic sinus surgery, septoplasty, posterior nasal neurectomy, and sinonasal tumor resection) were significantly lower than the preoperative scores (p < 0.001). Lee et al. [34] had reported that nasal surgery (septomeatoplasty, submucous resection of the septum, inferior turbinate resection, and endoscopic sinus surgery) can effectively reduce OSA patients' daytime sleepiness and snoring. Our findings provide empirical evidence that the benefits of office procedure LPN3 extend beyond relieving local nasal symptoms to improving systemic sleep outcomes. The significant reduction in PSQI scores indicates that relieving nasal obstruction minimizes sleep fragmentation and micro‐arousals caused by increased upper airway resistance [35]. This improvement in nocturnal sleep quality directly translated to functional benefits during the day, as evidenced by the concomitant decrease in ESS scores. The correlation between these two metrics suggests that the LPN3 procedure breaks the vicious cycle of “poor nasal breathing leading to non‐restorative sleep,” effectively reducing daytime hypersomnolence and restoring patients' daily alertness. This study provides evidence of improved subjective sleep quality (PSQI) and reduced daytime sleepiness (ESS), but it does not demonstrate objective improvement in OSA parameters. LPN3 should not be considered a curative treatment for OSA, and further studies with post‐operative HST or polysomnography are required to evaluate objective sleep architecture changes.

Regarding safety outcomes, no major complications—specifically major posterior epistaxis, dry eye, facial numbness, nasal pain, or sinusitis—were observed in any patient across both cohorts. This confirms that the laser energy was confined strictly to the target area without damaging the surrounding sensory nerves or autonomic pathways. A previous study by Huang et al. reported elevated post‐operation epistaxis risk when a patient combines hypertension, obesity, and OSA in turbinate surgery, combined with laser PNNN. However, the current study did not find a related effect that may be attributed to a more precise intervention.

In terms of minor adverse events, a subset of patients experienced transient reactive rhinorrhea lasting a few days, which resolved spontaneously without intervention. Huang et al. [36] reported that up to 64% of patients undergoing combined turbinate surgery and PNNN experienced moderate‐to‐severe rhinorrhea within the first postoperative week. Interestingly, we observed a higher frequency of minor epistaxis in the sham control group compared to the active arm. This counterintuitive finding likely stems from the procedural differences regarding hemostasis. Both groups underwent identical preparation, including local anesthesia injections and endoscopic manipulation, which inevitably cause micro‐trauma and needle‐track bleeding. However, in the active group, the laser energy provided immediate thermal coagulation, effectively sealing these microvessels and injection sites. In contrast, the sham group received the mechanical trauma of injection and instrumentation but lacked the coagulative benefit of the laser.

It is worth noting that a notable responder rate was observed in the sham control group, a phenomenon frequently described in interventional rhinology trials [29]. In our cohort, the sham control group achieved a mean rTNSS reduction of −2.6 (95% CI, −3.4 to −1.8). This is notably higher than the historical sham mean of −1.91 reported in a recent meta‐analysis of rhinologic RCTs [29]. This strong placebo response led to an unexpectedly high 30% responder rate of 63.6% in the sham arm. Several factors may contribute to this observation. First, invasive placebo procedures are known to elicit stronger therapeutic expectations than non‐invasive controls. Second, the local anesthesia, along with needle puncture, used in the sham protocol implies a pharmacological effect from vasoconstrictors, which may provide transient relief [37]. At this traditional threshold, the high level of “placebo noise” obscured the statistical separation between groups, making it difficult to confirm the laser treatment's physiological efficacy in this specific study population. To better capture the substantial clinical benefit of LPN3 and differentiate it from the strong placebo effect, we explored a more stringent 50% improvement threshold. This “high‐tier” responder analysis successfully filtered out the placebo responders, demonstrating a statistically significant and clinically meaningful superiority of LPN3 over the sham procedure (65.1% vs. 31.8%, *p* = 0.011). Despite this elevated baseline in the control arm, the LPN3 group demonstrated a statistically superior improvement, reinforcing the true physiological efficacy of the laser neurolysis beyond these non‐specific effects.

This study has several limitations. First, the final enrollment (*n* = 66) fell short of the initial target (*n* = 120). However, a broader literature review suggests a theoretical requirement of only 51 patients based on pooled effect sizes. Furthermore, post‐hoc analysis confirmed robust statistical power (88.8%) given the significant primary outcome (*p* = 0.002). Second, while investigators were not blinded, detection bias was mitigated as the primary endpoint (rTNSS) was patient‐reported. The integrity of patient blinding was further corroborated by comparable intraoperative pain scores during anesthesia between the active and sham groups. Third, the study is limited by its single‐center status and short (3‐month) follow‐up. However, extended monitoring is ongoing, and long‐term results following unblinding will be reported in future studies. In addition, the pragmatic design—which did not strictly restrict perioperative medications or require routine allergy testing—introduces potential confounders but enhances real‐world external validity, supported by evidence that PNNN efficacy is independent of allergic status. Finally, technical limitations include the inherent variability in laser dosimetry due to probe positioning and the need for a larger sample size to statistically validate the sensitivity and specificity of our proposed PLNA detection technique.

## 5 Conclusion

This RCT confirms LPN3 as a safe and effective treatment for refractory chronic rhinitis, delivering significant symptom relief by 3 months. This laser PLNA detection technique mitigates bleeding risks without compromising outcomes, proving advantageous for patients with narrow anatomy. In addition, concurrent improvements in PSQI and ESS scores demonstrate significant benefits for sleep quality. LPN3 thus represents a robust, safe alternative to contact‐based modalities.

## Author Contributions

E.Y.W., Y.S.H., M.S.T., C.H.C., J.W.H., C.J.W., and C.Y.H. designed and coordinated the study. J.Y.L., E.Y.W., H.L.T., and C.Y.H. enrolled the study participants and conducted the surgery. C.Y.H., C.H.C., Y.L.H., and J.W.H. collected the clinical data and drafted the article. C.Y.H., J.Y.L., and H.L.T. contributed significantly to the writing of the manuscript and documenting of most cases. All authors critically revised the manuscript for important intellectual content. All authors have read and approved the final manuscript.

## Funding

The study was supported by the Ditmanson Medical Foundation Chia‐Yi Christian Hospital Research Program (R113‐056).

## Ethics Statement

The study was approved by the Ethics Committee of Chia‐Yi Christian Hospital (No: CYCH2024022).

## Consent

All participants provided written informed consent prior to enrollment. The study was conducted in accordance with the Declaration of Helsinki.

## Conflicts of Interest

The authors declare no conflicts of interest.

## Supporting information




**Video**: Demonstration of posterior lateral nasal artery detection and CO_2_ laser posterior nasal nerve neurolysis.

## Data Availability

The data supporting this study's findings are not publicly available because they contain information that could compromise the privacy of research participants. Still, they are available from the corresponding author (C.Y.H.) upon reasonable request.
